# Natural Killer Cells Regulate Pulmonary Macrophages Polarization in Host Defense Against Chlamydial Respiratory Infection

**DOI:** 10.3389/fcimb.2021.775663

**Published:** 2022-01-04

**Authors:** Lei Zhao, Jing Li, Xiaoqing Zhou, Qianqian Pan, Weiming Zhao, Xi Yang, Hong Wang

**Affiliations:** ^1^Laboratory of Basic Medical Science, Qilu Hospital of Shandong University, Jinan, China; ^2^Department of Pathogenic Biology, School of Basic Medical Sciences, Shandong University, Jinan, China; ^3^Department of Clinical Laboratory, The First Affiliated Hospital of Bengbu Medical College, Bengbu, China; ^4^Department of Respiratory, Laiwu Central Hospital, Jinan, China; ^5^Departments of Immunology and Medical Microbiology and Infectious Diseases, Max Rady College of Medicine, University of Manitoba, Winnipeg, MB, Canada

**Keywords:** NK cells, macrophage, lung, Chlamydia, microRNAs

## Abstract

NK cells and pulmonary macrophages both are important components of innate immunity. The interaction between NK cells and pulmonary macrophages during chlamydial infection is poorly understood. In this study, we explored the effect of NK cells on regulation of pulmonary macrophage function during chlamydial respiratory infection. We found that NK depletion led to polarization of pulmonary macrophages from M1 to M2 phenotype, and it is related to reduced miR-155 expression in lung macrophage. Using adoptive transfer approach, we found that the recipients receiving lung macrophages isolated from *C. muridarum*-infected NK-cell-depleted mice exhibited an increased bacterial load and severe inflammation in the lung upon chlamydial challenge infection when compared with the recipients of lung macrophages from infected isotype control antibody treated mice. Herein, the effects of NK cells on macrophage polarization were examined *in vitro*. We found that NK cells from chlamydial-infected mice (iNK) significantly induced M1 polarization compared to that from uninfected mice (uNK). Inhibition of miR-155 expression in macrophages reduced M1 polarization induced by iNK, while miR-155 over-expression enhanced it. Furthermore, neutralization of IFN-γ in the coculture system decreased the expression of miR-155 by macrophages, and resulted in weakened M1 polarization. The data indicates that NK cells promote M1 polarization through up-regulation of miR-155 in macrophages by producing IFN-γ during chlamydial infection, and NK-regulated macrophage polarization is functionally relevant to host defense against the infection.

## Introduction

*Chlamydia*, as an obligate intracellular bacterium, causes a wide variety of human and animal diseases including ocular, pulmonary, and genital infections (Brunham and Rey-Ladino, 2005). So far, vaccine against chlamydial infections is not available, and antibiotic treatment is far from effectively controlling the prevalence of chlamydial infection because of its asymptomatic feature (Bebear and de Barbeyrac, 2009). Thus, intervention strategies for prevention and control of chlamydial-related diseases are urgently required, and a better understanding of the underlying mechanisms involved in shaping a protective immune response is critical. So far, it is well known that Th1 responses and IFN-γ production are crucial for elimination and controlling chlamydial infection, while Th2 response may be associated with immunopathology (Wang et al., 1999; Li et al., 2008; Gondek et al., 2009).

Macrophage, as an important innate immune cell and antigen presenting cell (APC), participates in maintaining immune homeostasis and resisting pathogen infection (Benoit et al., 2008; Murray and Wynn, 2011). Macrophages are recruited to the infected tissues during chlamydial infections (Morrison and Morrison, 2000). Macrophages in infected lung can contain *Chlamydia muridarum* (*C. muridarum*) and be activated subsequently (Gracey et al., 2015). A limited number of *in vivo* studies explored the role of macrophages in chlamydial infection (Rothfuchs et al., 2004; Qiu et al., 2008; Prantner et al., 2009; Miyairi et al., 2011; Gracey et al., 2015). Macrophages depletion resulted in an increased morbidity, more serious inflammation and higher bacterial growth after *C. muridarum* or *Chlamydophila psittaci* (*C. psittaci*) infection (Miyairi et al., 2011; Gracey et al., 2015). *In vivo* adoptive transfer of macrophages into RAG-1^-/-^/IFN-γ^-/-^ mice resulted in effective control of chlamydial lung infection (Rothfuchs et al., 2004). These researches imply that macrophages can provide protective immunity against chlamydial infection, although the underlying mechanism is not yet fully illustrated. In contrast, macrophages may also involve in immunopathology during chlamydial infection by persistently secreting proinflammatory cytokine (Prantner et al., 2009). Macrophages display strong functional plasticity, which is largely determined by microenvironment factors. Polarization state of macrophage is the key to its different functions in steady state and diseases (Murray et al., 2014). M1 polarized macrophage is characterized by high levels of pro-inflammatory cytokines secretion, leading to enhanced microbicidal and tumoricidal capacities. Alternative activated M2 macrophage produces high levels of anti-inflammatory cytokines and displays diminished ability to clear pathogens (Kahnert et al., 2006). It had been reported that *in vitro* chlamydial infection triggered pro-inflammatory M1 phenotype (Duncan et al., 2020), and M1 macrophages restricted the growth of *C. trachomatis*, while M2 macrophages were more suitable to support this (Tietzel et al., 2019). Whether the state of macrophage polarization is associated with infection outcome *in vivo* and how it is regulated during chlamydial infection are largely unknown.

Studies focused on the role of NK cell in chlamydial infection suggest that immunoregulation is the mainly mechanism carried out by NK cells in defending chlamydial lung infection instead of directly killing the pathogen (Jiao et al., 2011; Shekhar et al., 2015; Zhao et al., 2020). Our previous reports found that NK cells modulated Th1, Th17, Treg and T memory responses during chlamydial infection (Li et al., 2016; Wang et al., 2020; Zhao et al., 2020). The effect on T cell responses was mainly dependent on regulating dendritic cell (DC) function by NK and DC interaction (Jiao et al., 2011; Shekhar et al., 2015; Zhao et al., 2020). It has been reported that activation of macrophage in lung after chlamydial infection correlated with recruitment of IFN-γ-positive NK cells (Gracey et al., 2015), suggesting the possibility of the interaction between NK and macrophage. At present, there are few reports involved in regulation of macrophage function by NK cells. Depleted NK cells *in vivo* reduced the percentage of M1 macrophages in lung (Wu et al., 2020) and in visceral adipose tissue (Wensveen et al., 2015). In Mycobacterium tuberculosis infection model, macrophages shifted to M2 phenotype in the absence of IFN-γ-produced NK cells in T cell defective mice (Feng et al., 2006). In addition, NK cells killed pathogen-activated macrophages, resulting in restriction of excessive inflammation (Vankayalapati et al., 2005). Thus, NK cells may interact with macrophages to improve host resistance to intracellular bacterial infection.

MicroRNAs (miRNAs) are a class of endogenous non-coding RNAs that negatively regulate gene expression by mediating gene silencing. MicroRNAs have emerged as regulators of immune cell biological characteristics and inflammation (Mehta and Baltimore, 2016). It has been found that a variety of miRNAs can regulate the transcription of key molecules in macrophage polarization (Liu and Abraham, 2013). In chlamydial infection model, whether NK cells affect macrophage function and whether some miRNAs involve in this process have yet to be investigated. In the current study, we used NK depletion strategy to explore the impact of NK cell on lung macrophage function following chlamydial infection. More importantly, we demonstrated that NK cell influenced miRNAs expression of lung macrophages to regulate its polarization, mainly though producing IFN-γ.

## Materials and Methods

### Mice

Male BALB/c mice were purchased from Vital River Laboratories (Beijing) and maintained in a specific pathogen-free animal care facility of Shandong University. All mice used in this study were males between 6 and 8 weeks old. The animal experiments were conducted in compliance with the guidelines issued by the China Council for Animal Care and Utilization Committee of Shandong University, China (Permit Number: MECSDUMS2012056).

### Chlamydia and Mice Infection

*C. muridarum* organisms (Nigg strain) were cultivated, purified, and quantified as described (Joyee et al., 2007). The purified EBs were suspended in SPG buffer and stored at -80°C. The same seed stock of EBs was used throughout this study. For mouse infection, 1×10^3^ inclusion-forming units (IFUs) of live *C. muridarum* organisms in 40 μl SPG buffer were used to inoculate mice intranasally. Body weight of each mice was monitored daily. The growth of the organisms *in vivo* was determined as described previously (Yang et al., 1996). Briefly, the lungs were aseptically collected and homogenized in 3 ml sucrose phosphate glutamic acid buffer. Lung homogenates were centrifuged at 1,900 g for 30 min at 4°C, and the lung tissue supernatants were obtained and stored at -80°C. Hela 229 cell monolayers were incubated with tissue supernatants for 48 hours and fixed with absolute methanol. Inclusions were stained with a chlamydia genus-specific monoclonal antibody (provided by Professor Xi Yang, Department of Immunology and Department of Medical Microbiology, Faculty of Medicine, University of Manitoba) and a goat anti-mouse IgG conjugated to horseradish peroxidase (abcam, USA) as second antibody. The number of inclusions was counted under a microscope at X200 magnification and calculated based on dilution titers.

### NK Cell Depletion *In Vivo*

We used polyclonal anti–asialo-gangli-N-tetraosylcer-amide antibody (anti–asialo GM1) (Wako, Japan) to deplete NK cells in all experiments, which had been previously described (Li et al., 2016). Briefly, mice received an intravenous tail-vein injection of 20 μl anti-asialo GM1 or control normal rabbit IgG antibody (isotype) in 80 μl PBS 1 day before and 1 day after *C. muridarum* infection, then every 3 days injected 10 μl anti–asialo GM1 or isotype in 90 μl PBS until the end of the test. NK-cell depletion in the lung was confirmed by flow cytometric analysis.

### Lung Macrophage Purification and Adoptive Transfer

Single lung cell suspensions were obtained as described previously (Li et al., 2016). Briefly, lungs were cut into small pieces and digested with collagenase XI (2 mg/ml) (Sigma-Aldrich). The cell pellet was resuspended in 5 ml 35% (v/v) Percoll solution (Sigma-Aldrich) and centrifuged at 2000 rpm for 12 min at room temperature. Lung macrophage were sorted as CD45+F4/80+CD11b+cells using BD FACSMelody (BD Biosciences). For adoptive transfer, the purified lung macrophages from *C. muridarum* -infected NK-intact or NK-depleted mice were inoculated intranasally into naïve recipient mice (2×10^5^ Mφs/mouse). Two hours after the adoptive transfer, mice were intranasally inoculated with 1×103 IFUs of *C. muridarum* in 40 µl of PBS for challenge infection.

The sorted lung macrophages were at a concentration of 5×10^6^ cells per ml RPMI complete medium cultured with heat-inactivated *C. muridarum* (10^5^ IFU/ml) for 72 h. The supernatants were collected and analyzed for M1(TNF-α and IL-6)and M2 (IL-10) cytokines by ELISA.

### NK Cell Purification

Lung cells from either *C. muridarum*-infected mice or uninfected mice were prepared, and NK cells were isolated by negative selection using an NK cell isolation kit (Miltenyi Biotec) and magnetic column according to the manufacturer’s instructions. As determined by the flow cytometric analysis, the purity of NK cells was more than 95%.

### NK Cell and Macrophage Coculture

RAW264.7 cells (1×10^6^) were plated in 24-well plates overnight. On next day, purified NK cells (5×10^5^) from either *C. muridarum*-infected mice or uninfected mice were added in the presence of UK-EB (1×10^4^ IFUs/ml) for 24 h. Positive controls for M1 phenotype were generated by the addition of LPS (100 ng/ml) in RAW264.7 cells. For *in vitro* IFN-γ blocking experiments, anti-IFN-γ antibodies were added to co-cultures at 2 μg/ml, with rat IgG1κ as isotype control antibody. After 24h of co-culture, NK cells were removed though three times washing using PBS. Then, RAW264.7 cells were collected and its RNA was prepared for qPCR of indicated markers.

### Flow Cytometry

To examine the purity of lung macrophages and the population of M1/M2 cells after *C. muridarum* lung infection, freshly isolated lung mononuclear cells from NK-depleted and NK-intact mice were stained with anti-CD45-percp-cy5.5, anti-CD11b-FITC, anti-F4/80-PE, anti-iNOS-APC (M1) and anti-CD206-PE-cy7 (M2) antibodies (eBioscience). The raw data were collected using a BD FACSCanto 10C (BD Biosciences), and analyzed by Flowjo10.0 software.

### Histopathologic Analysis

For histopathologic analysis, the lung tissues were fixed in 10% buffered formalin and embedded in paraffin. The tissue sections were stained with hematoxylin and eosin, and the histopathologic changes and cellular infiltration were assessed by light microscopy. Histopathology score was carried out according to a set of custom designed criteria, which had been previously described (Horvat et al., 2007). The examiner was blinded as to the experimental groups.

### MicroRNA Microarray Analysis

The microRNA profiles of isolated NK+Mφs and NK-Mφs were investigated by Affymetrix miRNA 4.0 Array. In brief, macrophages were isolated and total RNA from each sample (n=3 per group) was extracted and quantified using the NanoDrop 2000C. The sample preparation and microarray hybridization were performed based on Arraystar’s standard protocols by Capitalbio Technology Corporation (Beijing, China).

### Real-time quantitative PCR (qPCR)

Total miRNA or mRNA was extracted from mouse lung macrophages and RAW264.7 cells by using Trizol reagent (Invitrogen), according to the manufacturer’s protocol. RNA quality was confirmed by electrophoresis on 1% agarose gel. RNA quantification was performed using NanoDrop™ One/OneC (Thermo Scientific), with optical density at 260 nm and 280 nm used to estimate the concentration and purity of total RNA. Approximately 1 μg of total RNA were conducted by gDNA Eraser (Takara) to remove the epibiotic gDNA. After the reaction, the concentration and purity of RNA were tested by NanoDrop™ One/OneC, which has little influence on the quality of RNA. The complementary DNA was synthesized by using the Mir-X™ miRNA qRT-PCR SYBR^®^ Kit or Primescript RT reagent kit (Takara). Real-time quantitative PCR involved use of the SYB green qPCR Mix (Takara) in a CFX96 real-time PCR instrument (Bio-Rad, USA). Each sample was run in triplicate. The sequences of mRNAs and miRNAs primers used for qPCR amplification were list in **Supplementary Data** (**Supplementary Tables 1**, **2**). The relative mRNA or miRNAs expressions were normalized to GAPDH or U6 level.

### Western Blot Assay

Lung tissues or macrophages were collected and immediately homogenized in solubilizing buffer at 4°C (1% Triton X-100,100 mM Tris-HCl, pH 7.4, 100 mM sodium pyrophosphate,100 mM sodium fluoride, 10 mM EDTA, 10 mM sodium orthovanadate, 2.0 mM PMSF, and 0.1 mg aprotinin/mL). Insoluble material was removed by centrifugation for 20 minutes at 9000g at 4°C. The protein concentration of the supernatants was determined by the Bradford assay. The samples were subjected to SDS-PAGE in a Bio-Rad (Hercules, CA) miniature slab gel apparatus (Mini-Protean). For immunoblot experiments, 0.2 mg of protein extracts from each tissue were separated by SDS-PAGE, transferred to PVDF membranes, and blotted with anti- iNOS, Arg-1, IRF5, IRF4 and GAPDH (abcam, USA). Proteins were visualized by chemiluminescence with an ECL kit (Millipore Corp., Billerica, MA, USA) and an enhanced chemiluminescence system (Amersham Life Science, Arlington Heights, IL, USA). The relative protein expressions were normalized to GAPDH. Band intensities were quantitated by optical densitometry (Scion Image software, ScionCorp, Frederick, MD).

### MicroRNA Transfection

MiR−155 mimics, miR−155 inhibitor and negative controls (NC) with random sequences were synthesized by Shanghai Genechem Co., Ltd. Mouse lung macrophages were seeded at a density of 5×10^5^ per well, and miR-155 inhibitor, mimics or NC were transfected after Lipofectamine 2000 treatment (Invitrogen, USA). Transfection was carried out for 18h followed by *C. muridarum* challenge. Then cells were harvested by trizol at 24h post infection, the total RNA was extracted as the manufacturer’s instructions (TAKATA, Japan).

### Statistical Analysis

All experiments were independently repeated at least 3 times. Statistical analysis involved use of Graphpad Prism 7 (Graphpad Software, San Diego, CA). Data are presented as mean ± SD. Unpaired Student’s t test or one-way ANOVA was used to determine significant differences between different groups. p<0.05 was considered statistically significant.

## Results

### NK Cell Depletion Redirect Macrophage Polarization During Chlamydial Lung Infection

**Figure 1:**
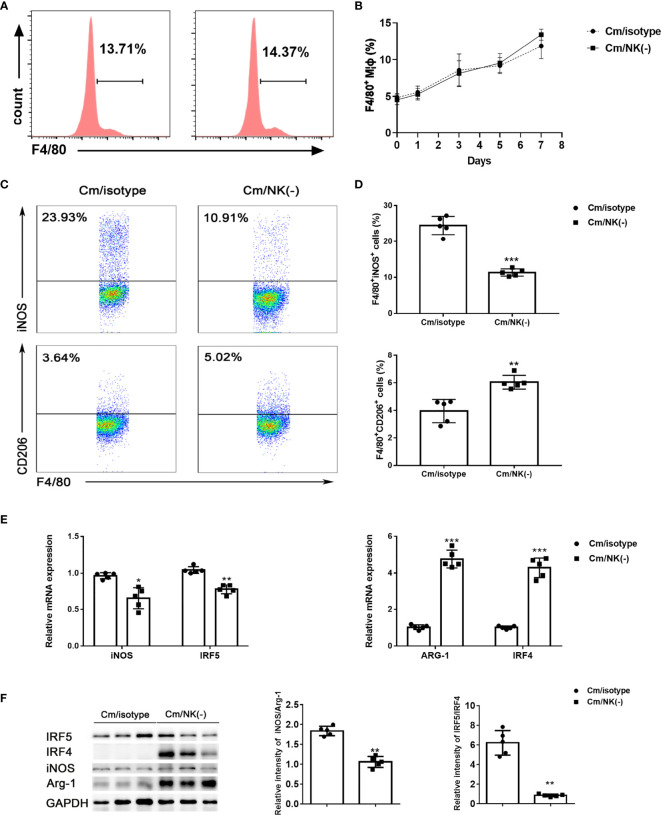
NK depletion reduced M1 type polarization following *C. muridarum* lung infection. BALB/c mice were infected with *C. muridarum* (1×10^3^ IFUs) intranasally, and treated with anti-asiola GM1 antibody or control antibody (isotype) before and during infection. Then, mice were killed at the indicated days, and lung single cells were analyzed by flow cytometry. **(A)** The representative histography were displayed for F4/80 expression gated on CD45 positive cells at day 7 p.i. **(B)**The percentage of macrophages (F4/80+cells) in the lung cells of each group at different time points of infection were shown. **(C)** Representative dot plots showing staining of iNOS+ or CD206+ macrophages of each group. **(D)** Flow cytometry data were summarized to show the percentage of iNOS+ (upper panel) or CD206+ (lower panel) macrophages in the different groups. **(E)** Lung macrophages were sorted by flow cytometry. The mRNA levels of iNOS, ARG-1, IRF5 and IRF4 in lung macrophages were measured by qPCR. **(F)** Western blot analysis and relative protein levels for iNOS, ARG-1, IRF5 and IRF4 in lung tissue lysates. At least three independent experiments with 5 mice in each group were performed, with one representative experiment is shown. Data are shown as mean± S.D. *p < 0.05; **p < 0.01; ***p < 0.001.

In order to examine the impact of NK cells on pulmonary Mφs, we depleted NK cells *in vivo* by intraperitoneal injection of anti–asialo-GM1 antibody and examined macrophage accumulation and polarization after *C. muridarum* lung infection. CD45+F4/80+CD11b+ cells were gated and analyzed as lung MΦs. We found that the percentage of Mφs were comparable in the lung between NK-depleted mice and isotype control antibody-treated mice following the infection (**Figures 1A, B**). Then, we analyzed macrophage polarization state by flow cytometry. As shown in **Figures 1C, D**, the percentage of M1(F4/80+iNOS+) population in the lung of NK-depleted mice was significantly decreased compared to that of isotype control mice. At the same time, the percentages of M2 (F4/80+CD206+) macrophage in NK cell-depleted mice was significantly increased. To further confirm the polarized state of lung macrophages, we sorted these cells from NK-depleted mice (NK-Mφs) and control mice (NK+Mφs) *via* flow cytometry sorting, and total RNA was extracted to perform qPCR on M1- and M2-specific genes. As expected, NK-Mφs displayed increased expression of Arg1 and IRF4 compared to NK+ Mφs, while the expressions of iNOS and IRF5 by NK- Mφs were decreased (**Figure 1E**). Furthermore, macrophage polarization in presence or absence of NK cells was also confirmed by the ratio of iNOS/Arg-1 and IRF5/IRF4 protein expression. As shown in **Figure 1F**, NK-Mφs showed decreased iNOS/Arg-1 and IRF5/IRF4 ratio at protein levels compared to NK+ Mφs.

Taken together, these findings suggested that NK cell deficiency in *C. muridarum*-infected mice results in shift of macrophage polarization to alternatively activated M2 phenotype, which may weaken the ability of macrophage to control the infection.

### Macrophages From NK-Depleted Mice Displayed Altered Cytokine Profiles

**Figure 2:**
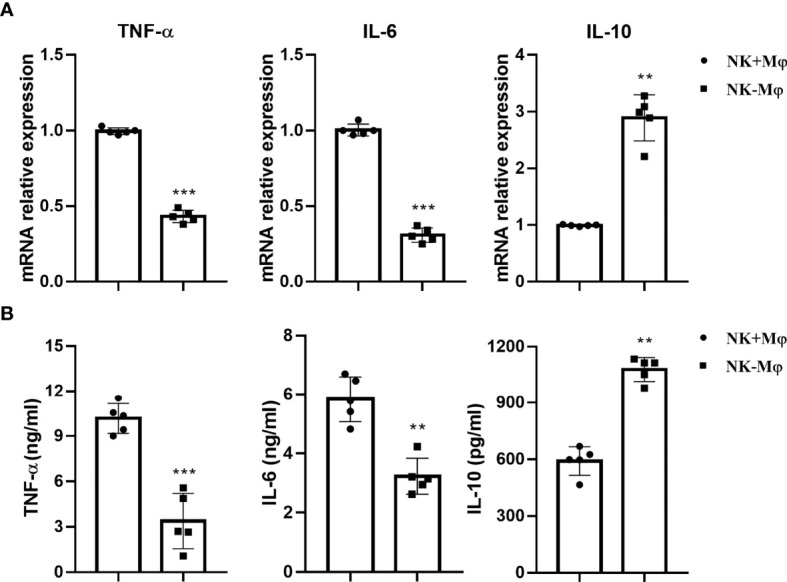
NK depletion altered cytokine production of macrophages. Lung macrophages were isolated from *C.muridarum*-infected NK-intact (NK+Mφs) and NK-depleted mice (NK-Mφs) at day 7 p.i. **(A)** The mRNA expression of TNF-α, IL-6 and IL-10 by NK+Mφs and NK-Mφs. **(B)** The protein levels of cytokines in the supernatants of sorted NK+Mφs and NK-Mφs were assayed by ELISA. Data are shown as mean ± S.D (n=5) and are representative of three independent experiments with similar results. **p < 0.01; ***p < 0.001.

The mRNA expression of TNF-α, IL-6 and IL-10 by NK+Mφs and NK-Mφs were analyzed by qPCR. The results showed that the expression of TNF-α and IL-6 in NK-Mφs was significantly lower than NK+Mφs after *C. muridarum* infection, while the mRNA expression of IL-10 was significantly increased in NK-Mφs (**Figure 2A**). Then, the cytokine production by purified pulmonary Mφs was detected by ELISA in supernatants, which confirmed the mRNA data (**Figure 2B**). These findings indicated that NK cells modulated cytokine production pattern of pulmonary Mφs by enhancing the production of TNF-α and IL-6 as well as inhibiting IL-10 secretion during chlamydial infection.

### Macrophages From NK-Depleted Mice Displayed Reduced Ability to Generate Protection Against *C. muridarum* Challenge Infection

**Figure 3:**
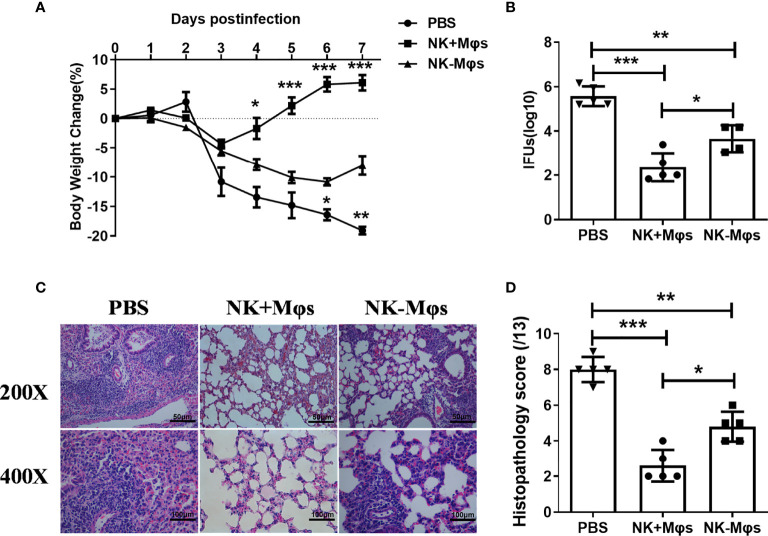
Adoptive transfer of NK+Mφs and NK-Mφs to estimate their function *in vivo*. NK+Mφs and NK-Mφs were adoptive transferred to naïve recipients. Mice receiving PBS only were used as control. After transfer, mice were challenged with 1×10^3^ IFUs of *C. muridarum*. **(A)** The percentage of body weight changes in the three groups of mice. **(B)** The chlamydial loads in lung at day 7 post challenge infection. The mean of log10 transformed IFUs per lung is presented. **(C)** The pathology of lung tissue. The lung tissue sections were routinely stained with H&E and observed in ×200 and ×400 magnification under light microscopy. **(D)** The score of tissue inflammatory grades. One-way ANOVA was used to determine significant differences between different groups. (n=5) *P < 0.05, **P < 0.01, ***P < 0.001.

To examine whether the macrophage phenotypic conversion caused by NK cell depletion play a role in macrophage-mediated antibacterial immunity, we conducted adoptive transfer experiments with NK-Mφs and NK+Mφs, and compared their ability to induce protective immunity against *C. muridarum* challenge infection. NK+Mφs or NK-Mφs were intranasally transferred to naïve recipient mice, then the mice were challenged with intranasal *C. muridarum* infection. As shown in **Figure 3A**, the mice receiving NK+Mφs showed much lower body weight loss and faster recovery compared to mice receiving only PBS with the same challenge infection. Although NK-Mφs also provided protection against the infection to some extent compared with PBS group, the mice receiving NK-Mφs were much less protected, showing higher body weight loss compared to mice receiving NK+Mφs. Consistently, the bacterial burden in the lung of NK+Mφs recipients is significantly lower than NK-Mφs recipients and PBS control (**Figure 3B**). Adoptive transfer of NK+Mφs considerably reduced pathologic changes in the lung of recipient mice, showing no obvious exudate in the alveolar cavity and patchy peribronchial cellular infiltrates. Meanwhile, receiving NK-MФs only mildly mitigated the pathological changes in recipients (**Figures 3C, D**). These data indicated that NK cell deficiency reduced the ability of Mφs in controlling *C.muridarum* infection.

### NK Cells From *C. muridarum*-Infected Mice Induced Macrophage Polarization Into M1 Phenotype *In Vitro*

**Figure 4:**
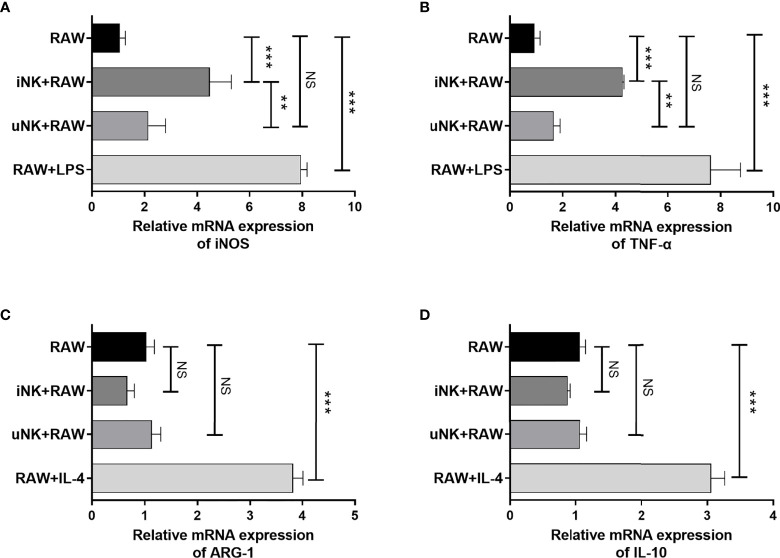
NK cells from *C.muridarum*-infected mice polarized macrophages toward an M1 phenotype *in vitro*. NK cells from lung of *C.muridarum*-infected (iNK) or uninfected mice (uNK) were co-cultured with RAW264.7 cells at a 1 to 2 ratio for 24h. After removing NK cells from the culture, iNOS **(A)**, TNF-α **(B)**, ARG-1 **(C)** and IL-10 **(D)** gene expression were analyzed in macrophages by qPCR. Data are shown as mean ± S.D and are representative of three independent experiments with similar results. One-way ANOVA was used to determine significant differences between different groups. (n=5) NS, no significant difference; **p < 0.01; ***p < 0.001.

To examine directly modulating effect of NK cells on macrophage polarization and possible underlying mechanism, an ex vivo co-culture experiment of RAW264.7 cells with spleen NK cells was used. We purified NK cells from *C. muridarum*-infected mice (iNK) at day 3 p.i. or uninfected mice (uNK), and cocultured them with RAW264.7 cells in the present of UK-inactivated *C. muridarum*. LPS induced M1 polarization and IL-4 induced M2 polarization were taken as control. As shown in **Figures 4A, B**, iNK effectively enhanced M1 polarization, as shown by increased iNOS and TNF-α expression by macrophage, while uNK had no significant effect. Meanwhile, M2 polarization markers Arg-1 and IL-10 in macrophage cocultured with either iNK or uNK were much lower than IL-4-treated RAW264.7(**Figures 4C, D**).

### MiR-155 Mediate Macrophage Polarization Modulated by NK Cells During Chlamydial Lung Infection

**Figure 5:**
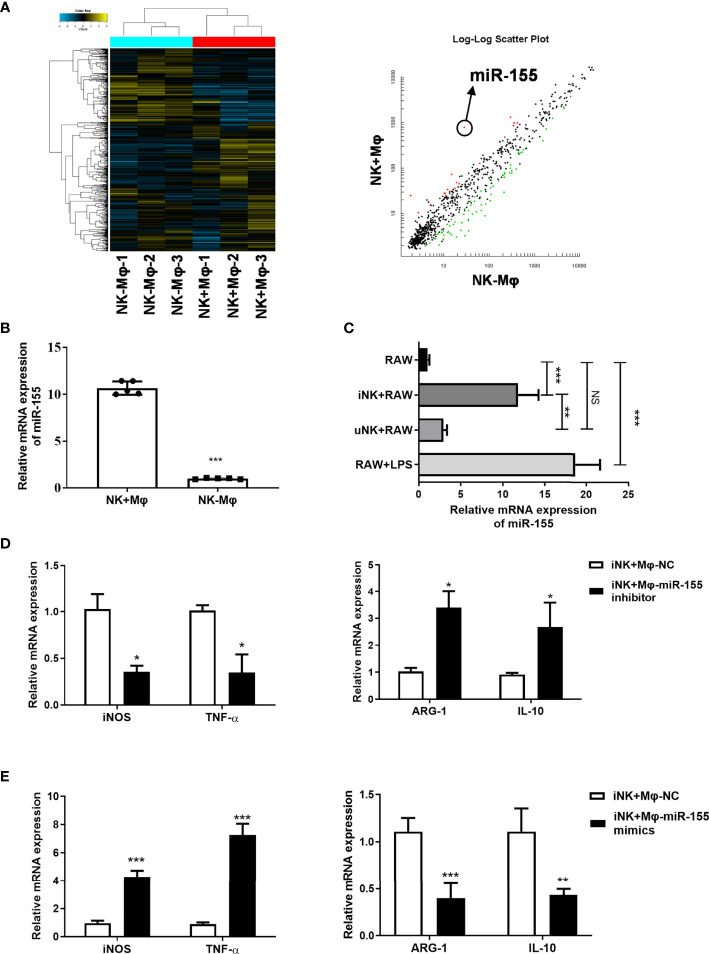
Targeting miR-155 affected macrophage polarization induced by NK cells from *C.muridarum*-infected mice (iNK). **(A)** The miRNAs profile of lung isolated NK+Mφs and NK-Mφs were conducted by microarray; The miR-155 showed significant difference between the two groups. **(B)** The miR-155 expression levels in isolated NK+Mφs and NK-Mφs. **(C)** The miR-155 expression in RAW264.7 cells in ex vivo NK and macrophage interaction. (iNK, NK *C.muridarum*-infected mice; uNK, NK cells from uninfected mice) **(D)** RAW264.7 cells were transfected with miR-155 inhibitor or control inhibitor and cultured with iNK. The gene expression of iNOS, TNF-α, ARG-1 and IL-10 were analyzed in macrophages by qPCR. **(E)** RAW264.7 cells were transfected with mimics of miR-155 or a respective control miRNA and cultured with iNK. The gene expression of iNOS, TNF-α, ARG-1 and IL-10 were analyzed in macrophages by qPCR. Representative data of three independent experiments with similar results are shown. Values are expressed as mean ± S.D. (n=5) NS, no significant difference; *p < 0.05; **p < 0.01; ***p < 0.001.

To explore the possible mechanisms by which NK cells polarize macrophages, we focus on microRNAs which were previously reported to be involved in macrophage polarization (Li et al., 2018). In the microarray-based analysis for miRNAs, we observed a marked upregulation of miR-155 in NK+Mφs compared to NK-Mφs (**Figure 5A**).

MiR-155 have been reported to be involved in regulation of macrophage polarization (Liu and Abraham, 2013). We verified miR-155 expression in isolated NK+Mφs and NK-Mφs at day 7 p.i. by qPCR. The result showed that miR-155 expression in NK-Mφs was significantly lower compared to NK+Mφs (**Figure 5B**). Then, we evaluated the changes of miR-155 expression in RAW264.7 cells which were cocultured with NK in ex vivo experiment. In consistent with *in vivo* data, remarkable upregulation of miR-155 expression was shown in RAW264.7 cells in the present of iNK cells compared to uNK, although miR-155 expression induced by iNK was lower than that induced by LPS (**Figure 5C**). These results suggested that miR-155 might play a role in NK-regulated macrophage polarization during Chlamydial lung infection.

To determine whether miR-155 play a role in NK-promoted M1 polarization, RAW264.7 cells were transfected with miR-155 inhibitor or control inhibitor and cocultured with iNK cells. The effects of miR-155 manipulation on the expression of M1 markers (iNOS and TNF-α) and M2 markers (Arg-1and IL-10) were examined by qPCR. As shown in **Figure 5D**, compared with control inhibitor, transfection with miR-155 inhibitor significantly reduced the expression of iNOS and TNF-α, while middling increased Arg-1. In addition, miR-155-overexpressing RAW264.7 cells incubated with iNK showed mildly increased iNOS and TNF-α decreased Arg-1 (**Figure 5E**). MiR-155 manipulation had no effect on IL-10 expression. The data suggested that miR-155 plays an important role in promoting M1 polarization induced by *C. muridarum*-activated NK cells.

### IFN-γ Derived From NK Cells Promoted miR-155 Expression on Macrophages and Facilitated M1-Like Activation

**Figure 6:**
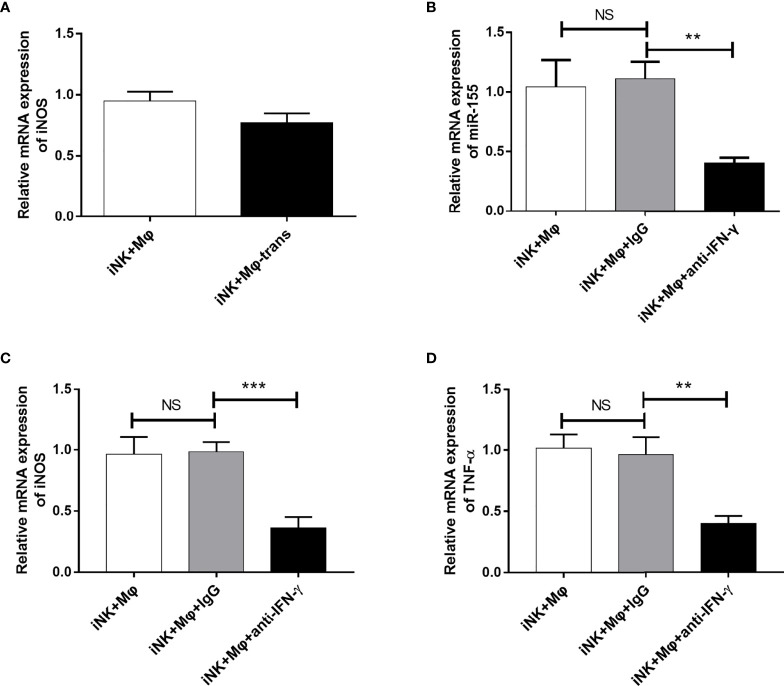
IFN-γ blocking inhibited miR-155 expression and M1 polarization in RAW264.7 cells. **(A)** NK cells from *C.muridarum*-infected mice (iNK) were cultured with or separated with RAW264.7 cells in transwell chamber. After 24h, iNOS gene expression by RAW264.7 cells were analyzed by qPCR. NK cells from *C.muridarum*-infected mice (iNK) were co-cultured with RAW264.7 cells at a 1 to 2 ratio for 24h in presence or absence of IFN-γ blocking antibody and IgG control antibody. Then, NK cells were removed from culture. **(B)** The miR-155 expression of RAW264.7 cells were shown. The iNOS **(C)** and TNF-α **(D)** gene expression were displayed. Representative data of three independent experiments with similar results are shown. Data are shown as mean ± S.D. (n=5) NS, no significant difference; **p < 0.01; ***p < 0.001.

To explored which factors derived from NK cells involved in M1 polarization in coculture system, we first blocked the physical contact between NK cells and RAW264.7 using Transwell chambers. The separation only slightly reduced the enhancing effect of NK cells on M1 polarization (**Figure 6A**). The data demonstrate that soluble proteins/factors released by NK cells may play major role in macrophage and NK interaction. IFN-γ is one of the important cytokines produced by *C. muridarum*-activated NK cells (Jiao et al., 2011). In order to examine the effect of NK cell-derived IFN-γ on RAW264.7 polarization, we carried out blocking experiment. Neutralization of IFN-γ activity by anti-IFN-γ antibody dramatically reduced miR-155 expression (**Figure 6B**). The iNOS and TNF-α expression were significantly decreased when IFN-γ was blocked, suggesting a predominant role of IFN-γ in modulation of M1 polarization by *C. muridarum*-activated NK cells (**Figures 6C, D**). The results provide direct evidence that NK cells activated by chlamydial infection can promote M1 polarization, and IFN-γ is critically important for the modulating effect of NK cells on macrophage polarization.

## Discussion

In this study, we employed the role of NK cells in regulation of lung macrophages during *C. muridarum* infection in both *in vitro* and *in vivo* systems. We found that NK cells had a significant promoting effect on the function of pulmonary macrophage, as shown by enhancing M1 polarization and pro-inflammatory cytokine production by macrophages, which eventually improved the protection against *C. muridarum* challenge infection induced by macrophages transfer. Further analysis showed that M1 polarization inhibited by NK depletion was related to decreased expression of miR-155 in lung macrophages. Using ex vivo coculture system, we further confirmed the role of miR-155 and IFN-γ in NK and macrophage interaction. Altogether, our findings shed light on the ability of NK cells to regulate the polarization of macrophages in controlling chlamydia infection.

The most important finding in the study is the promoting role of NK cells in directing M1 polarization of lung macrophages following chlamydial infection. Our previous reports had demonstrated the promoting effect of NK cells on Th1 and Th17 responses, and a significant inhibitory impact on Tregs response during chlamydial infection (Li et al., 2016). In this study, we focused on the immunoregulatory effect of NK cells on lung macrophage function in chlamydial infection. We found that NK cells promoted M1 polarization of lung macrophages and further enhanced their ability to generate protection against *C. muridarum* lung infection. This is supported by the findings showing;1) NK depletion resulted in a significantly altered macrophage polarization induced by *C. muridarum* infection (**Figure 1**), as shown by decreased iNOS but increased CD206 expression by lung macrophages. Moreover, the reduced ratios of iNOS/Arg-1 and IRF5/IRF4 as well as lower IL-6 and TNF-α production by isolated macrophage further supported weaker M1 polarization in NK-depleted mice. 2) In *in vitro* coculture system, NK cells isolated from chlamydia-infected mice induced M1 phenotype shift in mouse macrophage cell line RAW264.7 partially through IFN-γ production, but NK cells from naïve mice could not. It had been reported recently that *C. muridarum* infection induced macrophage polarization towards M1 phenotype (Duncan et al., 2020). M1 macrophages produce bactericidal substances and pro-inflammatory cytokines, which can directly kill intracellular pathogens and promote local Th1 cell responses. Without NK cells, the lung macrophage showed more M2-like phenotype (**Figures 1**, **2**), which may facilitate chlamydia growth (Tietzel et al., 2019). Indeed, our *in vivo* adoptive transfer experiment confirmed that NK-Mφs could not provide effective protection against *C. muridarum* challenge infection compared with NK+Mφs. The bacterial load in NK-Mφs recipients was much higher than in NK+Mφs recipients, indicating uncontrolled chlamydial growth in the lung (**Figure 3**). Our results support that macrophages, M1 macrophages in particular, are critical for generation of protective immune responses during chlamydial infection. It has been reported that M1-polarized macrophages may play a role in pathologic injury development during chlamydial infection (Harvie et al., 2019). In contrast with it, we did not observe increased pathological damage in NK+Mφs recipients. The discrepancy between our data and the reported finding may be associated with the difference of chlamydial model used in the studies. Together, the effect of NK cells on promoting M1 polarization is an important immunoregulatory mechanism in host defense against chlamydial lung infection.

M1/M2 polarization requires the coordination of a variety of specific transcription factors and intracellular signaling molecules (Murray et al., 2014). Studies have shown that miRNAs can regulate the key transcription molecules which are pivotal in macrophages polarization (Liu and Abraham, 2013). NK depletion significantly altered the expression of a large amounts of miRNAs by lung macrophages (GSE183577, https://www.ncbi.nlm.nih.gov/geo/query/acc.cgi?acc=GSE183577). Through analysis of several significantly different miRNAs expression in the NK+Mφs and NK-Mφs which had been reported to relate with macrophage polarization (data not shown), we speculated a possible association of miR-155 with the high M1 polarization in NK+Mφ. Previously published reports have shown that miR-155 has the ability to skew macrophages toward the M1 phenotype though targeting polarization-related transcription factors (Martinez-Nunez et al., 2011; Arranz et al., 2012). Our data showed that the expression of miR-155 was much lower in NK-Mφ than in NK+Mφ after chlamydial infection (**Figure 5**), suggesting possible role of miR-155 in lung macrophage polarization modulated by NK depletion. Indeed, in our *in vitro* NK and macrophage coculture experiment, NK cells from *C. muridarum*-infected mice enhanced miR-155 expression and M1 polarization in RAW264.7, and manipulation of miR-155 expression altered macrophage phenotype. MiR-155 has been reported to be required for Th1-type responses and the polarization of T cells towards a pro-inflammatory phenotype (Rodriguez et al., 2007). Similar to its function in T cell differentiation, miR-155 promotes macrophage polarization toward M1 type, which is known to facilitate the generation of Th1-type response. Together, miR-155 can tune in type 1 immune response though regulating M1 polarization and directly inducing Th1 differentiation, therefore it may play a role in regulation of anti-chlamydial protective immunity. Indeed, the enhanced susceptibility to chlamydia infection in miR-155-/- mice have been reported recently (Keck et al., 2020), but the underlying mechanism is largely unknown. The target genes of miR-155 need to be identify during NK and macrophage crosstalk in the context of chlamydial infection. In our infection model, we detected increased expression of SOCS1, a target of miR-155, in lung macrophage of NK cell-depleted mice compared to control mice (data not shown). SOCS1 has been shown as a negative regulatory molecule of M1 polarization (Nakagawa et al., 2002). Further study needs to be done to confirm whether SOCS-1is the direct target of miR-155 in the process of NK-regulated M1 polarization in response to chlamydial infection.

We sought to investigate which factors promoted miR-155 expression in iNK and macrophage interaction. The separation of iNK and macrophage by a 0.4 μm Transwell filter slightly reduced the effect of NK cells on macrophage polarization, indicating the major role of soluble factors. IFN-γ is a major effective factor of NK cells and also important cytokine to induce M1 polarization. It is not surprising that IFN-γ derived from chlamydial-activated NK cells is critical for NK and macrophage interaction, proven by IFN-γ blocking experiment. In addition, IFN-γ neutralization significant reduced miR-155 expression by macrophage, suggesting the regulatory role of IFN-γ on miR-155 expression during NK and macrophage interaction. The promoting effect of IFN-γ on miR-155 expression had been reported in macrophage functional study when macrophages were stimulated by TNF-α (Yee et al., 2017).

In summary, NK cells play an important immunomodulatory role in pulmonary macrophage polarization in Chlamydial lung infection. NK cells promote the shift of M1 phenotype and inhibit the M2 polarization by influencing the expression of miRNAs in macrophages, and the enhanced M1 polarization helps to control bacterial growth and reduce pathologic damage. This study extends the understanding of the protective role of NK cells in chlamydial infection and provides new insights into the interactions between innate immune system members in infectious diseases. The mechanism by which NK cells regulate the expression of miRNAs and subsequent intracellular signaling molecules in lung macrophages need to be explored. Our current study only observed the changes in miRNA expression of lung macrophages by depleting NK cells, *in vivo* injection of miRNAs inhibitors or mimics will be more conducive to obtain direct proof of miRNAs on regulating macrophage polarization. In addition, the strategies of NK cell depletion available for now in mouse models have limitations to some extent,the conclusion achieved by a study using specific strategy need to be interpreted dialectically. More future studies are needed to address these questions.

## Data Availability Statement

The datasets presented in this study can be found in online repositories: GSE183577, https://www.ncbi.nlm.nih.gov/geo/query/acc.cgi?acc=GSE183577. Other raw data supporting the conclusions of this manuscript will be made available by the authors, without undue reservation, to any qualified researcher.

## Ethics Statement

The animal study was reviewed and approved by China Council for Animal Care and Utilization Committee of Shandong University, China (Permit Number: MECSDUMS2012056).

## Author Contributions

HW and WZ designed the research study. JL and LZ performed the research experiments. XZ and QP analysed data. LZ and JL drafted the manuscript. XY and HW reviewed and edited the manuscript. All authors contributed to the article and approved the submitted version.

## Funding

The project was supported by National Natural Science Foundation of China (nos. 81501761, 81271853, 81902078, 30811120425) and Project ZR2020MH303 supported by Natural Science Foundation of Shandong Province.

## Conflict of Interest

The authors declare that the research was conducted in the absence of any commercial or financial relationships that could be construed as a potential conflict of interest.

## Publisher’s Note

All claims expressed in this article are solely those of the authors and do not necessarily represent those of their affiliated organizations, or those of the publisher, the editors and the reviewers. Any product that may be evaluated in this article, or claim that may be made by its manufacturer, is not guaranteed or endorsed by the publisher.

## Abbreviations

EB, elementary body; IFU, inclusion-forming unit; p.i., post infection; SPG, sucrose-phosphate-glutamic acid; IM, interstitial macrophage; Mφ, macrophages; iNOS, inducible nitric oxide synthase; TNF, tumor necrosis factor.
