# Supplementary material for: Natural Killer Cells Regulate Pulmonary Macrophages Polarization in Host Defense Against Chlamydial Respiratory Infection
Source: Front Cell Infect Microbiol. 2022 Jan 4;11:775663. doi: 10.3389/fcimb.2021.775663 (PMC8764407; doi:10.3389/fcimb.2021.775663)
Supplement: Supplementary file 1 [file DataSheet_1.docx]

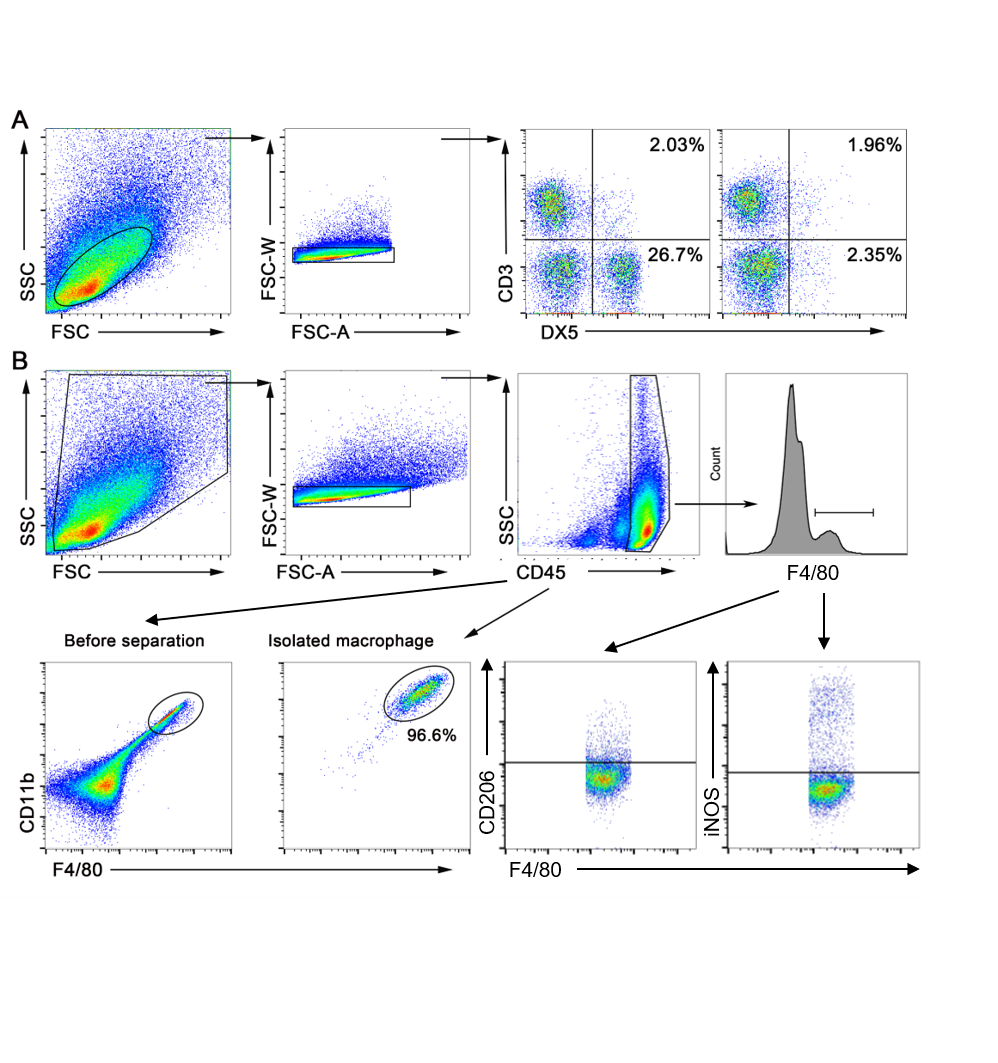


**Supplement figure 1.** The effect of NK depletion and flow cytometric gating strategies for macrophage analysis and purification. A. Flow cytometric images of NK cell (CD3-DX5+) in lung mononuclear cells from mice treated with anti–asialo GM1 or isotype antibody. B. The gating strategies for macrophage analysis and purification.
